# Microbial Contamination in the Coffee Industry: An Occupational Menace besides a Food Safety Concern?

**DOI:** 10.3390/ijerph192013488

**Published:** 2022-10-18

**Authors:** Carla Viegas, Bianca Gomes, Filipe Oliveira, Marta Dias, Renata Cervantes, Pedro Pena, Anita Quintal Gomes, Liliana Aranha Caetano, Elisabete Carolino, Ednilton Tavares de Andrade, Susana Viegas

**Affiliations:** 1 H & TRC—Health & Technology Research Center, ESTeSL—Escola Superior de Tecnologia e Saúde, Instituto Politécnico de Lisboa, 1990-096 Lisbon, Portugal; 2 Public Health Research Centre, NOVA National School of Public Health, Universidade NOVA de Lisboa, 1099-085 Lisbon, Portugal; 3 Comprehensive Health Research Center (CHRC), NOVA Medical School, Universidade NOVA de Lisboa, 1169-056 Lisbon, Portugal; 4 Department of Agricultural Engineering, Faculty of Engineering, Federal University of Lavras, Lavras 37203-202, Brazil; 5 Faculty of Medicine, Institute of Molecular Medicine, University of Lisbon, 1649-004 Lisbon, Portugal; 6 Research Institute for Medicines (iMed.uLisboa), Faculty of Pharmacy, University of Lisbon, 1649-003 Lisbon, Portugal

**Keywords:** milling stage, multi-approach for sampling and analyses, *Aspergillus*, azole resistance, One Health approach

## Abstract

Respiratory abnormalities among workers at coffee roasting and packaging facilities have already been reported; however, little is known about microbiological contamination inside coffee production facilities. This study intends to assess the microbial contamination (fungi and bacteria) in two coffee industries from Brazil with a multi-approach protocol for sampling and for subsequent analyses using four main sources of samples: filtering respiratory protection devices (FRPD) used by workers, settled dust, electrostatic dust cloths (EDC) and coffee beans. The fungal contamination in the assessed industries was also characterized through the molecular detection of toxigenic species and antifungal resistance. Total bacteria contamination presented the highest values in FRPD collected from both industries (7.45 × 104 CFU·m^−2^; 1.09 × 104 CFU·m^−2^). *Aspergillus* genera was widespread in all the environmental samples collected and sections with clinical relevance (*Fumigati*) and with toxigenic potential (*Nigri* and *Circumdati*) were recovered from FRPD. *Circumdati* section was observed in 4 mg/mL itraconazole. Sections *Circumdati* (EDC, coffee beans and settled dust) and *Nidulantes* (EDC, coffee beans and FRPD) were detected by qPCR. Some of the targeted *Aspergillus* sections that have been identified microscopically were not detected by qPCR and vice-versa. Overall, this study revealed that microbial contamination is a potential occupational risk in the milling stage and should be tackled when assessing exposure and performing risk assessment. In addition, a multi-sampling campaign should be the approach to follow when assessing microbial contamination and FRPD should be included in this campaign. Occupational exposure to mycotoxins should be considered due to high fungal diversity and contamination. A One Health approach should address these issues in order to prevent consumption of coffee crops and beans infected by fungi and, more specifically, to avoid widespread azole resistance.

## 1. Introduction

Coffee consumption has been increasing each year and coffee exports have amounted to 10.92 million bags in April 2021, compared with 11.24 million in April 2022. In fact, the export of coffee in the first 7 months of 2021/22 (21 October to 22 April) has increased by 0.6% [1]. However, we should bear in mind that climate change is also critically affecting the agricultural sector as plant growth is compromised but also toxigenic fungal growth, a major cause of plant death [2]. Thus, as with other crops, coffee that is one of the most traded commodities in the world is threatened by changing climate conditions and, consequently, by fungal infections [2].

Respiratory abnormalities among workers at coffee roasting and packaging facilities have already been reported [3,4,5,6,7] and exposure to dust, endotoxins, carbon monoxide, diacetyl, 2,3-pentanedione and other volatile organic compounds were previously assessed in coffee roasting facilities and coffees [8]. However, little is known about microbiological contamination inside coffee production facilities. In addition, since azole fungicides are largely applied in agriculture and material protection, fungi can come into contact with azoles everywhere. Thus, “Hot spots”—a habitat in which fungal species are disseminated and exposed to a fungicidally effective azole at concentrations that are high enough to select for resistant individuals potentially multiplying and spreading to other habitats—have already been identified by assessing the resistance risk in several occupational environments prioritizing those that handle food commodities. The coffee industry is one of these environments [9,10].

Mycotoxin occupational exposure should also be a concern since coffee beans are frequently contaminated with these fungal secondary metabolites. This happens due the crop infection by toxigenic fungi that commonly infect the plant during the various production stages (cultivation, processing or transport) [2,11]. As coffee requires wet conditions, the rainfall and humidity in areas for cultivation create the ideal conditions for *Aspergillus* species to grow, as these have optimal growth in warmer and humid climates [2,12]. Therefore, in an increasingly warmer world, mycotoxin production will increase, as higher temperatures and wetter climates provide perfect conditions for fungal growth and, consequently, mycotoxin production [2,12].

To our knowledge, data regarding occupational exposure to microbial contamination, obtained by a multi-approach strategy based on the use of different sampling methods and assays in coffee industries, have not been previously reported, and this omission has prevented risk management and control measures. Thus, this study intends to assess the microbial contamination (fungi and bacteria) in two coffee industries from Brazil with a multi-approach protocol for sampling and subsequent analyses using four main sources of samples: filtering respiratory protection devices used by workers, settled dust, electrostatic dust cloths and coffee beans. The fungal contamination in the assessed industries was also characterized through the molecular detection of toxigenic species and antifungal resistance.

## 2. Materials and Methods

### 2.1. Coffee Industries Characterization

**Figure 1:**
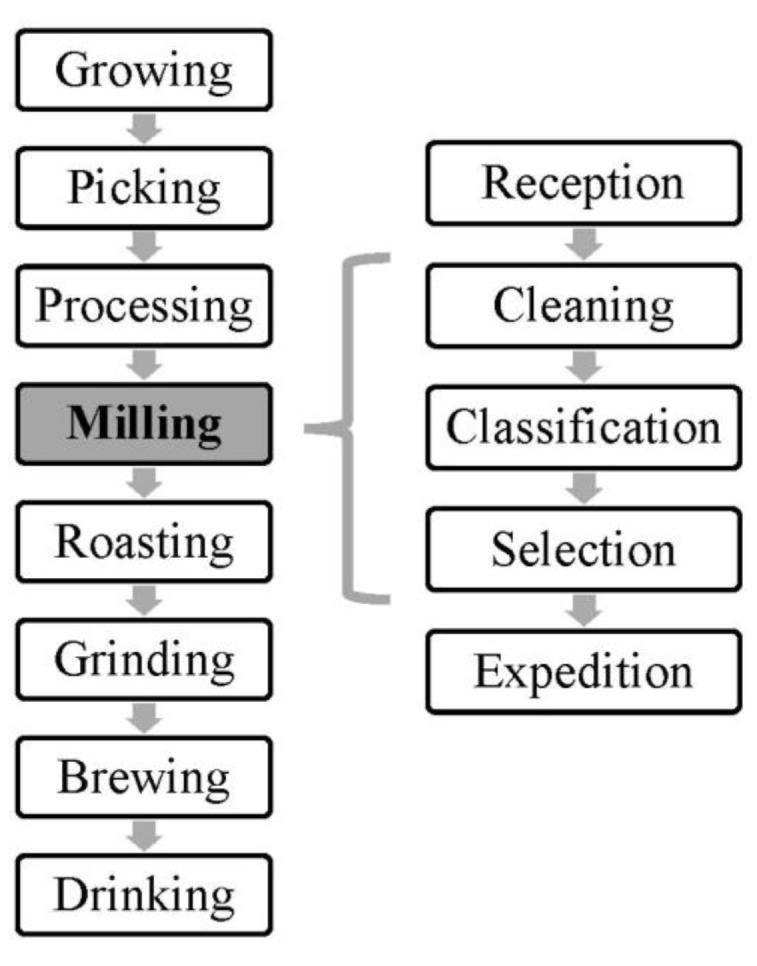
Coffee production flow.

The coffee industry involves different processes from the growth of the crops to the last step of being prepared for drinking. Typically, the main steps are growing, picking, processing, milling, roasting, packaging, shipping, grinding, brewing and drinking (Figure 1). Usually in Brazil, the country where samples have been collected, milling companies are the last stage before exportation. In Figure 1 we can see the common production flow, including all the steps that allow obtaining the final product on the coffee supply chain.

The samples were collected at two milling industries from two different mesoregions in Brazil: Campo das Vertentes e Sul and Sudoeste de Minas, which are within the largest producers of the country. According to the Brazilian Geographic and Statistics Institute (IBGE) [13], both mesoregions together produced 985,577 tons of coffee in 2017, which represents 27% of the national production.

Two industries—A and B—were sampled. Industry A has 11 workers and Industry B has 35 workers per shift (44 h per week), all working in warehouses from each industry. Regarding personal protection equipment used by workers, it was reported that workers have available respiratory protection devices with FFP1 filters, with or without exhalation valves.

### 2.2. Sampling Campaign Performed

The samples have been collected during August 2021, which is coffee harvest time in Brazil. The workplaces assessed and the sampling methods used are described in Table 1. Electrostatic dust collectors (EDC), settled dust, coffee bean samples and filtering respiratory protection devices (FRPD) were used as passive sampling methods.

Table 1 presents descriptions of the processing of *C. arabica* grains, their subcategories, varieties and types of processing for the companies in each region described above. Industry A works with specific grains from each producer, and thus, when they arrive at the processing unit, the grains are processed separately in batches. Industry B works in a single production batch with grains from different producers. This is the reason why it was not possible to distinguish, for the samples from industry B, which varieties were present no the processing used in the post-harvest.

The workplaces assessed were reception, milling, storage and expedition. In reception and expedition, the main activities carried out are weighing the grains, unloading the grains into the hoppers, sampling the product for classification and water content, loading the grains into the bags and moving the product. In milling, the main activities that take place in the sector are the movement of the product, removal of sticks, stones, leaves and other impurities, and separation of grains by size, density and color. In storage, the main activities that occur are the movement of the already bagged product and its storage.

EDC were placed in the sampling areas 1.5 ± 0.5 m above the ground for 15 days. Settled dust samples were collected with a sterilized spoon to gather the accumulated dust in each workplace [14]. Green coffee beans (GCB) were collected according to their subcategory using the classification from the Brazilian Official Classification for Coffee (COB) [15] (Table 2). Filtering respiratory protection devices (FRPD) used by coffee industry workers were also collected from workers belonging to industry A [16]. All samples were kept refrigerated (0–4 °C) in sterilized bags preceding analysis.

### 2.3. Sample Extraction and Characterization of Viable Microbiota

Passive samples were washed with 0.1% Tween 80 saline (0.9% NaCl) solution (250 rpm, 30 min), as follows: 20 mL solution for EDC; 9.1 mL solution for 1 g of settled dust sample and coffee beans [17] and 10 mL for FRPD filters [16]. Extracts were maintained frozen (−80 °C) with glycerol (2.23 mL for each g of settled dust and coffee beans, and 1.25 for FRPD) prior analysis [14,16].

Sample extracts were inoculated (150 μL) in malt extract agar (MEA) supplemented with chloramphenicol (0.05%), dichloran–glycerol agar (DG18), tryptic soy agar (TSA) supplemented with nystatin (0.2%), and Violet Red bile agar (VRBA) were used for fungi (MEA and DG18, 27 °C, 5–7 days), mesophilic (TSA, 30 °C, 7 days) and Gram-negative (VRBA, 35 °C, 7 days) bacteria selectivity. Microbial contamination quantification was determined as colony-forming units (CFU) and CFU concentration (CFU·m^−2^·day^−1^/g^−1^/m^−2^)^−^) after plate incubation. Morphological identification of fungal species was carried out through notation of macro and microscopic characteristics [18] by a trained mycologist.

### 2.4. Antifungal Susceptibility Testing

The screening of azole-resistant fungi was firstly carried out by seeding 150 μL of the extracts of all passive samples (N = 128) on Sabouraud dextrose agar (SDA) (Frilabo, Maia, Portugal) supplemented with 4 mg/L itraconazole (ITZ), 2 mg/L voriconazole (VCZ), or 0.5 mg/L posaconazole (PSZ), adapted from EUCAST guidelines [19,20]. The controls used were *A. fumigatus* reference strain (ATCC 204305) as the negative control, and pan-azole-resistant *A. fumigatus* strain as the positive control, both provided by the National Health Institute Doctor Ricardo Jorge, IP. After incubation for 2–3 days at 27 °C, identification was performed as previously described for fungal assessment [14].

### 2.5. Molecular Detection of the Targeted Fungal Sections

*Aspergillus* sections were amplified by quantitative PCR (qPCR) in the 8.8 mL samples’ extracts used in this study [17]. First, we isolated fungal DNA from the samples using the ZR Fungal/Bacterial DNA MiniPrep Kit (Zymo Research, Irvine, CA, USA). Then we performed qPCR amplification using the CFX-Connect PCR System (Bio-Rad, Amadora, Portugal). Reactions were performed in a 20 μL final volume containing 1 × iQ Supermix (Bio-Rad, Amadora, Portugal), 0.5 μM of each primer, and 0.375 μM of TaqMan probe. qPCR conditions included a three-step reaction consisting of 40 cycles of denaturation at 95 °C for 30 s, annealing at 52 °C for 30 s, and extension at 72 °C for 30 s.

H_2_O was used as a negative control and DNA isolated from a reference strain was used as a positive control. The reference strains were kindly provided by the Reference Unit for Parasitic and Fungal Infections from the Department of Infectious Diseases, National Health Institute Doctor Ricardo Jorge, Lisbon, Portugal. All reference strains were sequenced for ITS, B-tubulin and Calmodulin.

### 2.6. Statistical Analysis

Data were analyzed using SPSS statistical software for windows, version 27.0. The results were considered significant at the 5% significance level. To test the normality of the data, the Shapiro–Wilk test was used. For the comparison of bacterial contamination, fungal contamination and fungal resistance, the Kruskal–Wallis test was used, since the assumption of normality was not verified and given the small size of the sample. To study the relationship between bacterial contamination, fungal contamination and fungal resistance, Spearman’s correlation coefficient was used, since the assumption of normality was not verified. To assess species diversity, Simpson and Shannon indices, given by $\mathrm{Shannon}\;\mathrm{Index}\;\left(H\right)=-\overset{s}{\underset{i=1}{\sum }}p_{i}\ln\left(p_{i}\right)$ and $\mathrm{Simpson}\;\mathrm{Index}\;\left(D\right)=\frac{1}{\sum_{i=1}^{s}p_{i}^{2}}$, were used, where *p_i_* is the proportion (n_i_/n) of individuals of one particular species found (n_i_) divided by the total number of individuals found (n).

## 3. Results

### 3.1. Viable Bacterial Contamination

In what concerns industry A, total bacteria contamination (measured by TSA) presented the highest values in FRPD (7.45 × 10^4^ CFU·m^−2^), followed by grains (2.20 × 10^4^ CFU·g^−1^), EDC (2.20 × 10^4^ CFU·m^−2^·day^−1^) and settled dust (1.17 × 10^3^ CFU·g^−1^). In VRBA media, EDC evidenced the highest Gram-negative counts among the matrices (4.5 × 10^5^ CFU·m^−2^·day^−1^). Grains and FRPD had similar values of Gram-negative bacteria (2.52 × 10^3^ CFU·g^−1^ and 2.00 × 10^3^ CFU·m^−2^, respectively), while a lower value was obtained from settled dust samples (1.17 × 10^3^ CFU·g^−1^).

**Figure 2:**
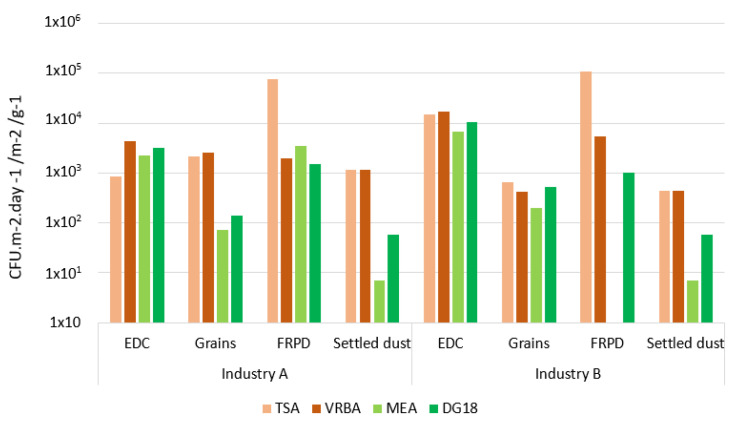
Bacterial (TSA; VRBA) and fungal (MEA; DG18) distribution among the sampled matrices (EDC: log [CFU·m^−2^·day^−1^]; Grains, settled dust: log [CFU·g^−1^]; FRPD: log [CFU·m^−2^]).Concerning fungal distribution, the highest fungal diversity was obtained from EDC samples on both coffee companies. Grain samples had the same diversity on samples isolated from both companies (MEA: 8 species; DG18: 5 species). In settled dust from industry B, five species were identified on MEA and seven species on DG18, while on industry A, three species were found on MEA and four species on DG18 in samples from the same matrix. Lower fungal diversity was associated with FRPD samples from industry A (MEA: five species; DG18: two species), whereas on industry B, two species were found on DG18.

Among all the analyzed matrices from industry B, the highest counts for total bacteria were found on FRPD (1.09 × 10^4^ CFU·m^−2^), while the second highest values were obtained from EDC (1.51 × 10^4^ CFU·m^−2^·day^−1^). The lowest values of total bacteria were obtained from grains (6.69 × 10^2^ CFU·g^−1^) and settled dust (4.40 × 10^2^ CFU·g^−1^) (Figure 2). Regarding VRBA media, the highest counts of gram-negative bacteria were found in EDC (1.69 × 10^4^ CFU·m^−2^·day^−1^), followed by FRPD (5.5 × 10^5^ CFU·m^−2^). Similar counts were obtained for settled dust and grains (4.40 × 10^2^ CFU·g^−1^ and 4.16 × 10^2^ CFU·g^−1^, respectively).

### 3.2. Viable Fungal Contamination

FRPD from industry A have evidenced the highest fungal counts (MEA: 3.50 × 10^3^ CFU·m^−2^; DG18: 1.50 × 10^3^ CFU·m^−2^) among all the collected samples, which was followed by EDC (MEA: 2.29 × 10^3^ CFU·m^−2^·day^−1^ DG18: 3.15 × 10^3^ CFU·m^−2^·day^−1^). The lowest counts were obtained on grains (MEA: 7.35 × 10^1^ CFU·g^−1^; DG18: 1.41 × 10^2^ CFUcfu·g^−1^) and settled dust samples (MEA: 7 CFU·g^−1^; DG18: 5.80 × 10^1^ CFU·g^−1^).

Regarding samples from industry B, the highest fungal contamination numbers were observed on EDC samples on both MEA and DG18 (6.73 × 10^3^ CFU·m^−2^·day^−1^; DG18: 1.06 × 10^4^ CFU·m^−2^·day^−1^ respectively), followed by grains (MEA: 2.01 × 10 CFU·g^−1^; DG18: 5.34 × 10^2^ CFU·g^−1^) and settled dust (MEA: 7 CFU·g^−1^; DG18: 5.80 × 10^1^ CFU·g^−1^). In contrast, no fungal counts were obtained in FRPD samples on MEA, only being identified on DG18 (DG18: 5.80 × 10^1^ CFU·m^−2^) (Figure 2).

Regarding species diversity on DG18, industry B was the one with higher diversity (Shannon index (H) = 1.23, Simpson index (D) = 2.83) (Table S1—Supplementary Material).

In what concerns industry A, the most common fungal genera observed in EDC samples was *Cladosporium* sp. on MEA (57.10%), and *Penicillium* sp. on DG18 (62.99%). The genera *Cladosporium* sp. was also frequent in grains on MEA (32.65), while on DG18, *Mucor* sp. was prevalent (92.20%). Regarding filters, *Paecilomyces* sp. and *Aspergillus* sp. were recurrent on MEA (42.86%), while on DG18, *Aspergillus* was the only genera identified (100%). In settled dust samples *Rhizopus* sp. was the dominant genera (71.43%), while on DG18, *Penicillium* sp. was prevalent (62.07%) (Table 3).

Among samples from industry B, *Penicillium* sp. was the most frequent fungal genera observed on EDC (32.35% MEA; 7.76% DG18). The same genus was prevalent on DG18 (66.48%) in grain samples, while on MEA, *Cladosporium* sp. was the most frequent (54.36%). *Aspergillus* sp. was the only genera found in FRPD on DG18 (100%), while *Rhizopus* sp. and *Penicillium* sp. were the most common genera identified in settled dust samples on MEA and DG18, respectively (Table 3).

**Figure 3:**
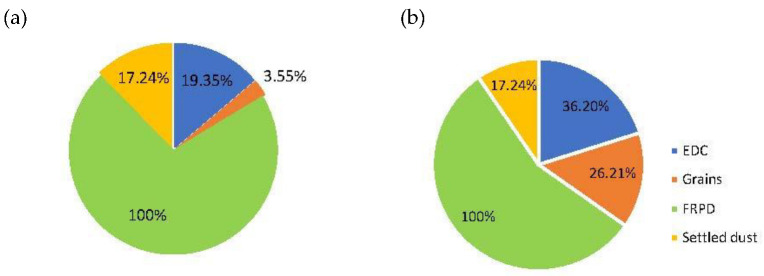
*Aspergillus* sp. distribution in DG18 culture medium in samples from: (**a**) industry A and (**b**) industry B. (EDC: log [CFU·m^−2^·day^−1^]; Grains, settled dust: log [CFU·g^−1^]; FRPD: log [CFU·m^−2^]).

Regarding *Aspergillus* sp., the highest value of this genera was obtained in samples incubated in DG18 from both industries (A: 43.83%; B: 40.92%) when compared to samples incubated with MEA (A: 27.73%; B: 20.01%). In samples from industry A, the most contaminated matrix with *Aspergillus* sp. genera were FRPD (42.86% MEA; 100.00% DG18), followed by settled dust (14.29% MEA; 17.24% DG18), grains (10.88% MEA; 3.55% DG18) and EDC (5.24% MEA; 19.35% DG18). Despite not having detected *Aspergillus* sp. on MEA, FRPD samples from industry B had the highest values of this fungal genera in DG18 (100%), followed by EDC (20.46% MEA; 36.20% DG18), settled dust (14.29% MEA; 17.24% DG18) and grains (4.99% MEA; 26.22% DG18) (Figure 3).

Concerning sections distribution on EDC from industry A, four *Aspergillus* sections were detected on MEA (3.70% *Nigri;* 0.93% *Circumdati*; 0.31% *Flavi;* 0.31% *Fumigati)* and six sections on DG18 (9.67% *Nigri*; 6.64% *Circumdati*; 1.57% *Fumigati*; 0.56% *Flavi*; 0.56% *Nidulantes*; 0.34% *Aspergilli*), while on FRPD, three sections were identified using MEA (14.29% *Circumdati*; *Fumigati*; *Nidulantes*) and two using DG18 (66.67% *Nigri*; 33.33% *Circumdati)*. Two sections were reported in the grain samples on MEA (5.44% *Restricti*; *Circumdati*) and on DG18 (2.13% *Circumdati*; 1.42% *Candidi*). *Aspergillus* section *Nidulantes* was dominant in the settled dust as detected on MEA (14.29%), while on DG18, two sections were identified (13.79% *Nigri*; 3.45 *Circumdati*).

**Figure 4:**
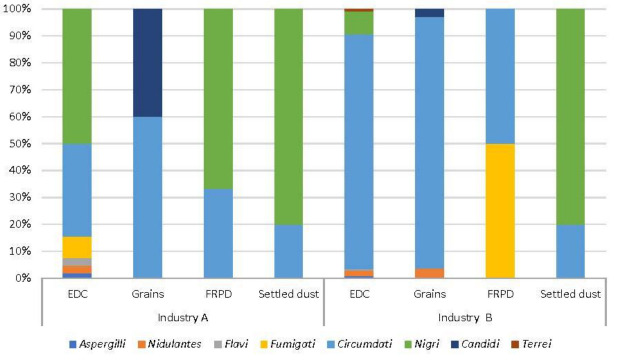
*Aspergillus* sections distribution in DG18 in both industries (EDC: log [CFU·m^−2^ day^−1^]; Grains, settled dust: log [CFU·g^−1^]; FRPD: log [CFU·m^−2^]).

Similar results were obtained in EDC samples from industry B, were four *Aspergillus* sections were reported on MEA (7.00% *Nigri*; 6.58% *Fumigati*; 6.58% *Circumdati*; 0.32% *Flavi*) and six sections on DG18 (31.58% *Circumdati*; 3.01 *Nigri*; 0.74% *Nidulantes*; 0.33% Terrei; 0.30% *Aspergilli*; 0.23% *Flavi*). On the grains, four sections were detected on MEA (2.49% *Circumdati*; 1.50% *Nidulantes*; 0.50% *Nigri*; 0.50% *Flavi*) and three sections on DG18 (24.53% *Circumdati*; 0.94% *Nidulantes*; 0.75% *Candidi*). In contrast to the prevalence of section *Nidulantes* in MEA (100%), two *Aspergillus* sections were identified in the settled dust on DG18 (13.79% *Nigri*; 3.45% *Circumdati*). In FRPD, two sections were detected in DG18 (50.00% *Circumdati* and 50.00% *Fumigati)* (Figure 4).

### 3.3. Fungal Diversity in Azole-Supplemented Media

**Figure 5:**
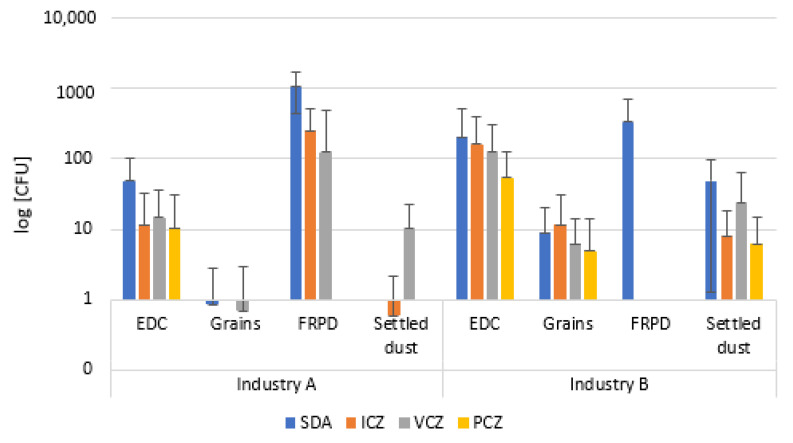
Fungal average counts, per industry and sample matrix (EDC, log CFU·m^−2^·day^−1^; Grains, settled dust, log CFU·g^−1^; FRPD, log CFU·m^−2^), by screening in azole-supplemented Sabouraud dextrose agar (SDA) media. ICZ, 4 mg/mL itraconazole; VCZ, 2 mg/mL voriconazole; PCZ, 0.5 mg/mL Posaconazole.

**Figure 6:**
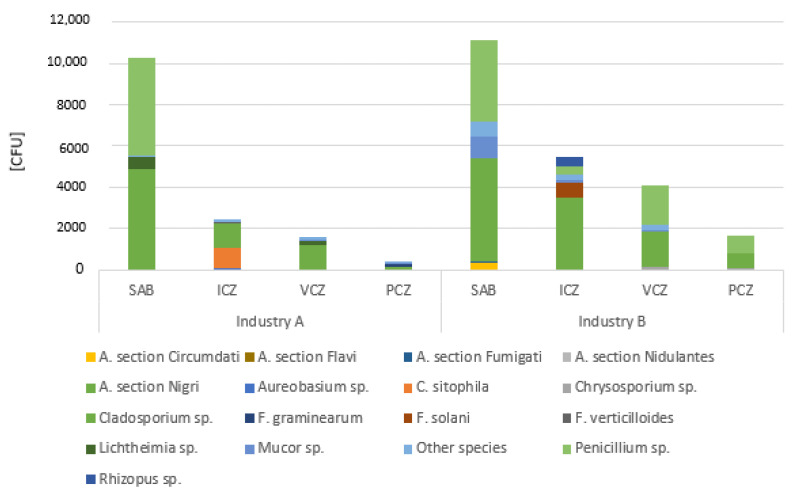
Fungal diversity, per industry, by screening in azole-supplemented Sabouraud dextrose agar (SDA) media. ICZ, 4 mg/mL itraconazole; VCZ, 2 mg/mL voriconazole; PCZ, 0.5 mg/mL posaconazole.

The highest average fungal counts were determined in FRPD and EDC (Figure 5). The most frequent fungi present in SDA and in azole-supplemented SDA media were *Cladosporium* sp. and *Penicillium* sp., with five different *Aspergillus* sections observed in SDA (Figure 6). In azole-supplemented media, only section *Circumdati* was observed, more specifically in 4 mg/L ICZ, recovered from one EDC sample in one coffee brand (Supplementary Material—Figure S1).

### 3.4. Detection of the Targeted Fungal Sections

*Aspergillus* section *Nidulantes* was detected in 20 out of 128 samples (15.6%), with 6 (4.7%) being present in EDC samples, 3 (2.3%) in coffee beans samples, 1 (0.8%) in FRPD samples and 10 (7.8%) in settled dust samples. Concerning *Aspergillus* section *Circumdati*, it was detected in 26 samples out of the 128 samples (20.3%), with 20 being detected in EDC samples (0.8%), 1 in coffee bean samples and 6 (4.7%) in settled dust samples (Supplementary Material—Table S2).

Despite not having detected *Aspergillus* in some samples, the genus was identified through culture-based methods in some matrices from industry A (two samples from EDC (2.5%); one from settled dust (5%) and one from FRPD (16.6%), and in industry B (one sample from EDC (1.6%); one from settled dust (5%) and one from coffee beans (6.25%)). In addition, *Aspergillus* section *Circumdati* was identified in industry A, more specifically in 14 EDC samples (17.5%), 1 settled dust sample (5%) and 3 types of coffee beans (15%). On the other hand, in industry B, *Circumdati* was observed in 11 EDC samples (18.3%), 3 settled dust samples (15%) and 7 coffee bean (4.4%) samples.

### 3.5. Comparisons and Correlation Analysis

Regarding bacterial contamination in TSA, statistically significant differences were detected between the sampling sites of the two companies (χ^2^(7) = 115.163, *p* = 0.000). It was found that FRPD and EDC from industry B, as well as FRPD from industry A, were the ones with the highest bacterial contamination in TSA. In VRBA, statistically significant differences were also detected between the sampling methods of the two companies (χ^2^(7) = 77.673, *p* = 0.000), and it was also observed that the EDC of industry B displayed the highest contamination load, followed by the settled dust and the grains of industry A (Supplementary Material—Figure S2).

Considering fungal contamination, statistically significant differences were detected between the sampling methods of the two companies, both in MEA (χ^2^(7) = 72.164, *p* = 0.000) and in DG18 (χ^2^(7) = 60.836, *p* = 0.000), having been observed (in both media) that the industry B’s EDC exhibited the highest contamination values (Supplementary Material—Figure S2).

With regard to fungal resistance, statistically significant differences were also detected between the sampling methods of the two companies in all the media applied, SDA (χ^2^(7) = 65.232, *p* = 0.000), ITZ (χ^2^(7) = 74.681, *p* = 0.000), VCZ (χ^2^(7)=58.673, *p* = 0.000) and PSZ (χ^2^(7) = 42.085, *p* = 0.000). The sampling methods that showed the highest values were the FRPD of industry A, followed by the EDC of industry B in SDA; the EDC of industry B followed by the FRPD of industry A in ITZ; industry B’s EDC were followed by industry B’s settled dust in VCZ; EDC and settled dust of industry B in PSZ (Supplementary Material—Figure S2).

Regarding the relationship between bacterial and fungal contamination and fungal resistance, the following significant correlations were detected: (i) greater bacterial contamination in TSA is related to greater bacterial contamination in VRBA, higher values of fungal resistance in SDA and ITZ; (ii) higher bacterial contamination in VRBA is related to higher fungal contamination in MEA and DG18 and higher values of fungal resistance in ITZ, VCZ and PSZ; (iii) higher fungal contamination in MEA is related to higher fungal contamination in DG18 and higher values of fungal resistance in SDA, ITZ, VCZ and PSZ; (iv) fungal contamination in DG18 is related to higher values of fungal resistance in SDA, ITZ, VCZ and PSZ; (v) higher values of fungal resistance in SDA are related to higher values of fungal resistance in ITZ, VCZ and PSZ; (vi) higher values of fungal resistance in ITZ are related to higher values of fungal resistance in VCZ and PSZ; (vii) higher values of fungal resistance in VCZ are related to higher values of fungal resistance in PSZ (Table 4).

## 4. Discussion

Several studies have previously reported both microbial [21,22,23,24,25,26,27,28,29,30] and mycotoxin contamination [25,27,30,31,32,33] in coffee grains. The later are essentially ochratoxin A, the only mycotoxin monitored so far in coffee production (green and roasted coffee) [34]. However, in what concerns occupational health, less attention has been given to exposure assessment in the coffee industry. Based on this concern, the International Labour Organization has recently published a toolkit for action to improve occupational safety and health in the coffee supply chain where microbiologic agents are referred to as a risk factor to be considered in this industry [35]. In fact, despite the potential contamination of coffee cherries and beans on the different phases of plant development and in all the supply chain, mainly with fungi and mycotoxins, most of the studies have only reported results of exposure to organic dust and workers’ respiratory health [3,36,37]. A deeper analysis concerning microbial contamination exposure assessment in coffee industry facilities has been lacking thus far.

To address this issue, in our study, a comprehensive protocol of passive sampling methods was applied in two coffee industries, A and B assessed. Concerning bacterial contamination, the same sampling method—FRPD—exhibited the highest counts in both industries, while on fungal contamination, different sampling methods presented the highest counts within each industry (FRPD on A; EDC on B). These results followed the same trend as in previous studies developed in other types of occupational environments, where sampling methods provided different results when applied to various workplaces [38,39,40]. Additionally, statistically significant differences were found between the different sampling methods in both industries for bacterial and fungal contamination. This situation reinforces the need to apply multiple sampling methods, thus avoiding a stand-alone sampling method approach [40]. As in previous studies performed in the waste sorting industry [41], FRPD proved to be a proper passive sampling method to be applied in comprehensive sampling campaigns to assess occupational exposure to fungal contamination. In fact, during FRPD use, suitable conditions for microorganisms’ growth can be provided (water vapor, humidity, temperature, etc.) potentiating workers exposure [42]. This was confirmed by the presence, in this study, of the highest counts of *Aspergillus* sp. on FRPD.

In this work, it was also possible to validate the microbial contamination as an occupational risk factor in this specific coffee industry occupational environment. In fact, Gram-negative bacteria was common in all environmental matrices analyzed also supporting the possible exposure to endotoxins in this setting, as previously reported in studies conducted in coffee industries [3,43]. Therefore, the exposure to bacteria in this setting, in particular, the exposure to specific species/strains and endotoxins should be seriously considered. Furthermore, it is known that bacteria viability on the analyzed protection devices (FRPD) can be increased [44], since this occupational environment is characterized by having high amounts of dust [3,36,37] allowing the transport of nutrients to be retained on the filtration material from FRPD [45,46]. In addition, fungal species with toxigenic potential [47] and clinical relevance [48] were observed and detected through all the different matrixes analyzed, emphasizing their presence in FRPD, that are in direct contact with workers´ respiratory airways, allowing a more real exposure scenario by inhalation and subsequent health effects [41]. *Aspergillus* section *Fumigati* presence on FRPD should be considered as a critical occupational risk, since inhaling its spores may cause several diseases (aspergilloma, invasive pulmonary aspergillosis and different forms of hypersensitivity diseases), depending on the immunological status of the exposed workers [49,50]. Overall, the obtained results corroborate the microbiologic contamination as an occupational risk and justify the inclusion of the FRPD in screening campaigns in order to achieve detailed exposure assessments [41].

As in previous studies performed by our team, *Aspergillus* sections were observed at higher counts on DG18 [14,17], due the feature of this media to restrict fungal species with fast-growing rates, such as those belonging to Mucorales order (*Mucor*, *Rhizopus* and *Lichtheimia* genera) [16].

In addition, the results clearly show the relevance of using different methods to detect and/or identify microorganisms in a given sample as some microorganisms that have been identified microscopically were not detected by qPCR and vice-versa. These differences between molecular and culture-based methods have been observed previously where the low growth speed of fungal species was pinpointed as the main reason for the lack of their detection in culture systems, on the one hand, and high spore prevalent fungi as being on the basis of preferential detection by molecular-based methods, on the other hand [51]. Importantly, qPCR allows the detection of toxigenic species which are not possible to distinguish microscopically [52]. The presence of the *Aspergillus* sections *Nidulantes* and *Circumdati* also identified by molecular biology tools, reveals an important contamination by potential toxigenic fungi. Their quantification would provide a better idea of the risk assessment exposure of each worker/working space. Still, it is possible, from the CT values, to identify areas with a higher degree of contamination, as a lower CT indicates the presence of a higher microorganism contamination.

As mentioned before, the main OTA producers were observed (*Aspergillus* sections *Circumdati* and *Nigri*) in all the environmental matrices and section *Flavi* was identified in both industries. Of most relevance is the observation of section *Flavi*, the main producer of aflatoxins (e.g., aflatoxin B1), classified by IARC as carcinogenic to humans (Group 1) [53]. These findings claim attention for the need to consider mycotoxin presence in this workplace environment [54,55]. Indeed, the coffee workplace environment is the ideal setting for this phenomena due to several factors, including (a) the fact that the raw material handle is prone to fungal contamination [23,29], (b) fungal species known as mycotoxin producers are detected (e.g., *Aspergillus* species), (c) high dust contamination due to manual tasks are performed in this setting (e.g., as storage work, loading, handling or milling) promoting high exposure to organic dust [36,56] that act as carriers of mycotoxins to the lungs [57,58] promoting exposure via inhalation [59,60,61,62,63], but also dermal absorption due to the deposition of dust particles containing mycotoxins in the skin. In addition, work surfaces contaminated with dust particles can also be considered opportunities for further skin contact and hand to mouth contact promoting exposure also by ingestion [64,65].

The widespread use of demethylation inhibitors (DMI) as fungicides in several economic sectors, such as agriculture, medicine, animal husbandry and material preservation, has led to the reduced efficacy of medical DMI antifungals used to treat patients infected with *Aspergillus fumigatus* due to the presence of azole-resistant isolates [66,67,68,69,70]. Due to their high efficiency and broad-spectrum activity, the DMI fungicides (which include triazoles and imidazoles) are the most used fungicides in many disease management programs to protect crops against fungal infections that compromise production yields [71,72,73]. They are particularly important in the protection of cereals, fruits, vegetables and other crops against fungal diseases, thus supporting food security.

Although in the present study, *Aspergillus* section *Fumigati* was not detected in azole-supplemented media by passive sampling (only *Circumdati* section was observed in the presence of 4 mg/mL itraconazole), the surveillance of azole resistance in crops and other environments is highly recommended. The crop protection industry strongly encourages the research on the potential for specific agricultural settings that are able to select and amplify azole-resistant *A. fumigatus*. The science-based, multisectoral One Health approach is of utmost importance to address this problem, by evaluating settings in which the selection of resistance mutations is plausible, and defining effective mitigation measures when necessary. In addition, it would be of relevance to identify mutations in these strains that can correlate with the azole resistance, namely in the case of *Aspergillus fumigatus*, due to its clinical relevance.

## 5. Conclusions

Overall, this study clearly suggests that the microbial contamination should be considered an occupational risk in the coffee industry (in this case, the milling stage) and should be tackled when assessing exposure and performing risk assessment. In addition, a multi-sampling campaign should be the approach to follow where FRPD analysis should be included.

The present study also draws attention to the need for considering occupational exposure to mycotoxins, in the milling stage, among others, due to high fungal diversity and contamination. Moreover, these workers are exposed simultaneously to fungi, bacteria and probably to their metabolites, an exposure scenario that brings several challenges concerning risk assessment and management.

A One Health approach applied to the coffee industry will address these issues through effective specific actions such as preventing coffee crops and beans from being infected by fungi and, more specifically, avoiding widespread azole resistance. This represents important challenges due to the climate change scenario that requires proper attention and accurate risk management measures.

## Figures and Tables

**Table 1:** Workplaces assessed and sampling methods applied.

Industry	Production	Number of Workers per Shift	Workplaces Assessed	Sampling Methods (n)				
				EDC	Settled Dust	Coffee Beans	FRPD	Observations (Photos from the Workplaces)
A		11	Reception/ Expedition	8	2	1	-	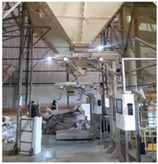
			Milling	22	8	2	4	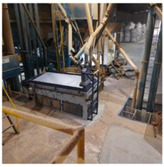
			Storage	10	10	7	4	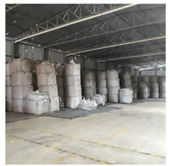
			Total	40	10	10	8	
B		35	Reception	2	1	-	n.s	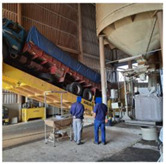
			Storage	2	2	-	n.s	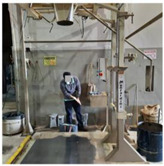
			Expedition	6	2	-	n.s	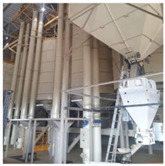
			Milling	20	5	8	n.s	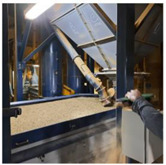
			Total	30	10	8	12	

n.s.: not specified; FRPD: Filtering respiratory protection devices.

**Table 2:** Green coffee beans sample description.

Industry	Samples	Category	Subcategory	Variety	Processing Type
A	1	*Cofeea arabica*	bica corrida	n.s.	n.s.
	2	*Cofeea arabica*	n.s.	n.s.	n.s.
	3	*Cofeea arabica*	n.s.	n.s.	n.s.
	4	*Cofeea arabica*	bica corrida	Catuaí/Catucaí	wet process
	5	*Cofeea arabica*	bica corrida	Catuaí/Catucaí	wet process
	6	*Cofeea arabica*	mocha	Bourbon/Catucaí	wet process
	7	*Cofeea arabica*	bica corrida	Catuaí/Catucaí	dry process
	8	*Cofeea arabica*	large flat	Bourbon	wet process
	9	*Cofeea arabica*	large flat	Bourbon/Catucaí	dry process
	10	*Cofeea arabica*	medium flat	Catuaí	wet process
B	1	*Cofeea arabica*	large flat	mixed	n.s.
	2	*Cofeea arabica*	large flat	mixed	n.s.
	3	*Cofeea arabica*	mocha	mixed	n.s.
	4	*Cofeea arabica*	medium flat	mixed	n.s.
	5	*Cofeea arabica*	large flat	mixed	n.s.
	6	*Cofeea arabica*	bica corrida	mixed	n.s.
	7	*Cofeea arabica*	bica corrida	mixed	n.s.
	8	*Cofeea arabica*	bica corrida	mixed	n.s.

n.s.: not specified.

**Table 3:** Fungal distribution per sampling method EDC: log [CFU·m^−2^ day^−1^]; Grains, settled dust: log [CFU·mg^−1^]; Filters: log [CFU·mm^−2^]).

	MEA			DG18		
INDUSTRY A						
Sample	Fungi	CFU· m^−3^/m^−2^/g^−^^1^/CFU·m^−2^·day^−1^	%	Fungi	CFU·m^−3^/m^−2^/g^−^^1^/CFU·m^−2^·day^−1^	%
EDC	*Cladosporium* sp.	1309.27	57.10	*Penicillium* sp. *Cladosporium* sp. *Aspergillus* sp. Other species	1981.60 346.78 608.63 208.78	62.99 11.02 19.35 6.64
	*Mucor* sp.	286.62	12.50			
	*Chrysosporium* sp.	208.78	9.10			
	*Aspergillus* sp.	120.31	5.25			
	Other species	368.01	16.05			
Grains	*Cladosporium* sp. *Penicillium* sp. *Chrysosporium* sp. *Aspergillus* sp. Other species	24.00 21.00 20.00 8.00 3.50	32.65 28.57 27.21 10.88 4.76	*Mucor* sp. *Penicillium* sp. *Aspergillus* sp. Other species	130.00 5.00 5.00 1.00	92.20 3.55 3.55 0.71
FRPD	*Paecilomyces* sp. *Aspergillus* sp. Other species	1500.00 1500.00 500.00	42.86 42.86 14.29	*Aspergillus* sp.	1500.00	100.00
Settled dust	*Rhizopus* sp. *Aspergillus* sp. *Penicillium* sp.	5.00 1.00 1.00	71.43 14.29 14.29	*Penicillium* sp. *Mucor* sp. *Aspergillus* sp.	36.00 12.00 10.00	62.07 20.69 17.24
**INDUSTRY B**						
EDC	*Penicillium* sp. *Cladosporium* sp. *Aureobasidium* *Aspergillus* sp. Other species	2176.22 2041.76 2075.73 1376.50 56.62	32.35 30.35 15.99 20.46 0.84	*Penicillium* sp. *Aspergillus* sp. *Cladosporium* sp. Other species	5046.00 3825.19 1641.90 53.08	47.76 36.20 15.54 0.50
Grains	*Cladosporium* sp. *Penicillium* sp. *Rhizopus* sp. *Aspergillus* sp. Other species	109.00 66.00 13.50 10.00 2.00	54.36 32.92 6.83 4.99 1.00	*Penicillium* sp. *Aspergillus* sp. *Cladosporium* sp.	355.00 140.00 39.00	66.48 26.22 7.30
FRPD	*-*	-	-	*Aspergillus* sp.	1000.00	100.00
Settled dust	*Rhizopus* sp. *Aspergillus* sp. *Penicillium* sp.	5.00 1.00 1.00	71.43 14.29 14.29	*Penicillium* sp. *Mucor* sp. *Aspergillus* sp.	36.00 12.00 10.00	62.07 20.69 17.24

**Table 4:** Study of the relationship between bacterial and fungal contamination and fungal resistance. Results of Spearman correlation coefficient.

		Bacteria	Fungi		Fungal Resistance			
		VRBA	MEA	DG18	SDA	ITZ	VCZ	PSZ
Bacteria	TSA	0.322 **	0.020	−0.053	0.300 **	0.219 *	0.003	−0.002
	VRBA		0.357 **	0.303 **	0.038	0.444 **	0.391 **	0.342 **
Fungi	MEA			0.649 **	0.448 **	0.627 **	0.573 **	0.506 **
	DG18				0.289 **	0.592 **	0.504 **	0.382 **
Fungal resistance	SDA					0.551 **	0.335 **	0.339 **
	ITZ						0.686 **	0.615 **
	VCZ							0.584 **

** Correlation is significant at the 0.01 level (2-tailed). * Correlation is significant at the 0.05 level (2-tailed).

## Data Availability

Not applicable.

## Supplementary Materials

The following supporting information can be downloaded at: https://www.mdpi.com/article/10.3390/ijerph192013488/s1, Table S1: Shannon and Simpson diversity indices in the EDC matrix; Figure S1: *Aspergillus* sections’ frequencies, per industry (A, B) and sampling matrix (EDC, grains, settled dust), by screening in azole-supplemented Sabouraud dextrose agar (SDA) media. ICZ, 4 mg/mL itraconazole; VCZ, 2 mg/mL voriconazole; PCZ, 0.5 mg/mL posaconazole; Table S2: *Aspergillus* sections detection in the different matrices analyzed; Figure S2: Comparison of bacterial, fungal contamination and fungal resistance between the sampling methods of the two industries (A and B). Results of the Kruskal Wallis test.

## References

[1] ICO (2022). International Coffee Organization. https://www.ico.org/.

[2] Adhikari M., Isaac E.L., Paterson R., Maslin M.A. (2020). A Review of Potential Impacts of Climate Change on Coffee Cultivation and Mycotoxigenic Fungi. Microorganisms.

[3] Sakwari G., Mamuya S., Braatveit M., Larsson L., Pehrson C., Moen B. (2013). Personal Exposure to Dust and Endotoxin in Robusta and Arabica Coffee Processing Factories in Tanzania. Ann. Occup. Hyg..

[4] Bailey R., Cox-Ganser J., Duling M., LeBouf R., Martin S., Bledsoe T., Green B.J., Kreiss K. (2015). Respiratory morbidity in a coffee processing workplace with sentinel obliterative bronchiolitis cases. Am. J. Ind. Med..

[5] Duling M.G., LeBouf R.F., Cox-Ganser J.M., Kreiss K., Martin S.B., Bailey R.L. (2016). Environmental characterization of a coffee processing workplace with obliterative bronchiolitis in former workers. J. Occup. Environ. Hyg..

[6] Harvey R., Hawley B., Korbach E., Rawal A., Roggli V., Bailey R., Cox-Ganser J.M., Cummings K.J. (2018). Flavoring-related lung disease in a worker at a coffee roasting and packaging facility. Am. J. Respir. Crit. Care Med..

[7] Harvey R., Fechter-Leggett Ethan D., Bailey Rachel L., Edwards Nicole T., Fedan Kathleen B., Virji M.A., Nett Randall J., Cox-Ganser Jean M., Cummings Kristin J. (2020). The Burden of Respiratory Abnormalities Among Workers at Coffee Roasting and Packaging Facilities Frontiers. Public Health.

[8] Hawley B., Cox-Ganser J.M., Cummings K.J. (2017). Carbon monoxide exposure in workplaces, including coffee processing facilities. Am. J. Respir. Crit. Care Med..

[9] Gisi U., Deising H.B., Fraaije B., Mehl A., Oerke E.C., Sierotzki H., Stammler G. (2020). Resistance risk for DMI fungicides in Aspergillus fumigatus: Potential hot spots. Modern Fungicides and Antifungal Compounds IX.

[10] Viegas C., Caetano L.A., Viegas S. (2021). Occupational exposure to Aspergillus section Fumigati: Tackling the knowledge gap in Portugal. Environ. Res..

[11] Bennett J.W., Klich M. (2003). Mycotoxins. Clin. Microbiol. Rev..

[12] Peterson R.R.M., Lima N. (2010). How will climate change affect mycotoxins in food?. Food Res. Int..

[13] IBGE (2022). Brazilian Geographic and Statistics Institute. https://www.ibge.gov.br/en/home-eng.html.

[14] Viegas C., Gomes B., Pimenta R., Dias M., Cervantes R., Carolino E., Twarużek M., Soszczynska E., Kosicki R., Viegas S. (2022). Microbial contamination in Firefighter Headquarters: A neglected occupational exposure scenario. Buid. Environ..

[15] (2012). COB, Brazilian Official Classification for Coffee. Insight Special: Brazilian Coffees. https://volcafespecialty.com/wp-content/uploads/2017/10/April-12-IS-Brazilian-Coffees.pdf.

[16] Viegas C., Twarużek M., Dias M., Almeida B., Carolino E., Soszczyńska E., Viegas S., Aranha Caetano L. (2021). Cytotoxicity of filtering respiratory protective devices from the waste sorting industry: A comparative study between interior layer and exhalation valve. Environ. Int..

[17] Viegas C., Sousa P., Dias M., Caetano L.A., Ribeiro E., Carolino E., Twarużek M., Kosicki R., Viegas S. (2021). Bioburden contamination and Staphylococcus aureus colonization associated with firefighter’s ambulances. Environ. Res..

[18] De Hoog D., Guarro J., Gene G., Figueras M. (2016). Atlas of Clinical Fungi—The Ultimate Benchtool for Diagnosis.

[19] Arendrup M.C., Rodriguez-Tudela J.L., Lass-Flörl C., Cuenca-Estrella M., Donnelly J.P., Hope W. (2013). EUCAST technical note on anidulafungin. Clin. Microbiol. Infect..

[20] European Committee on Antimicrobial Susceptibility Testing (EUCAST) (2020). Routine and Extended Internal Quality Control for MIC Determination and Agar Dilution for Yeasts, Moulds and Dermatophytes as Recommended by EUCAST. http://www.eucast.org.

[21] Mislivec P.B., Bruce V.R., Gibson R. (1983). Incidence of toxigenic and other molds in non-ripe coffee beans. J. Food Prot..

[22] Nakajima M., Tsubouchi H., Miyabe M., Ueno Y. (1997). Survey of aflatoxin B1 and ochratoxin A in commercial green coffee beans by high-performance liquid chromatography linked with immunoaffinity chromatography. Food. Agric. Immunol..

[23] Silva C.F., Schwan R.F., Dias E.S., Wheals A.E. (2000). Microbial diversity during maturation and natural processing of coffee cherries of Coffea Arabica in Brazil. Int. J. Food Microbiol..

[24] Batista L.R., Chalfoun S.M., Prado G., Schwan R.F., Wheals A.E. (2003). Toxigenic fungi associated with processed (green) coffee beans (*Coffea arabica* L.). Int. J. Food Microbiol..

[25] Noonim P., Mahakarnchanakul W., Nielsen K.F., Frisvad J.C., Samson R.A. (2008). Isolation, identification and toxigenic potential of ochratoxin A-producing Aspergillus species from coffee beans grown in two regions of Thailand. Int. J. Food Microbiol..

[26] Rezende E., Borges J., Cirillo M., Prado G., Paiva L., Batista L. (2013). Ochratoxigenic fungi associated with green coffee beans (*Coffea arabica* L.) in conventional and organic cultivation in Brazil. Braz. J. Microbiol..

[27] Culliao A.G., Barcelo J.M. (2015). Fungal and mycotoxin contamination of coffee beans in Benguet province, Philippines. Food Addit. Contam. Part A Chem. Anal. Control Expo. Risk Assess..

[28] Casas-Junco P.P., Ragazzo-Sánchez J.A., Ascencio-Valle F.J., Calderón-Santoyo M. (2017). Determination of potentially mycotoxigenic fungi in coffee (*Coffea arabica* L.) from Nayarit. Food Sci. Biotechnol..

[29] Viegas C., Pacífico C., Faria T., De Oliveira A.C., Caetano L.A., Carolino E., Gomes A.Q., Viegas S. (2017). Fungal contamination in green coffee beans samples: A public health concern. J. Toxicol. Environ. Health A.

[30] Al Attiya W., Hassan Z.U., Al-Thani R., Jaoua S. (2021). Prevalence of toxigenic fungi and mycotoxins in Arabic coffee (*Coffea arabica*): Protective role of traditional coffee roasting, brewing and bacterial volatiles. PLoS ONE.

[31] Pitt J.I., Basílico J.C., Abarca M.L., López C. (2000). Mycotoxins and toxigenic fungi. Med. Mycol..

[32] World Health Organization, Food and Agriculture Organization (2001). Safety Evaluation of Certain Mycotoxin in Food.

[33] García-Moraleja A., Font G., Mañes J., Ferrer E. (2015). Analysis of mycotoxins in coffee and risk assessment in Spanish adolescents and adults. Food Chem. Toxicol..

[34] EU (2006). European Comission, Food Safety—Ochratoxin A Legislation. https://ec.europa.eu/food/safety/chemical-safety/contaminants/catalogue/ochratoxin_en.

[35] ILO (2021). International Labour Organization. Improving Occupational Safety and Health in the Coffee Supply Chain. https://vzf.ilo.org/wp-content/uploads/2021/07/Toolkit_VZF_web_compressed.pdf.

[36] Abaya W.S., Bråtveit M., Deressa W., Kumie A., Moen B. (2018). Personal Dust Exposure and Its Determinants among Workers in Primary Coffee Processing in Ethiopia. Ann. Work Exp. Health.

[37] Magne B.M., Wakuma A.S., Gloria S., Bente E. (2021). Dust Exposure and Respiratory Health Among Workers in Primary Coffee Processing Factories in Tanzania and Ethiopia. Public Health Front..

[38] Viegas C., Dias M., Viegas S. (2022). Electrostatic Dust Cloth: A Useful Passive Sampling Method When Assessing Exposure to Fungi Demonstrated in Studies Developed in Portugal (2018–2021). Pathogens.

[39] Viegas C., Pena P., Dias M., Gomes B., Cervantes R., Carolino E., Twarużek M., Soszczyńska E., Kosicki R., Caetano L. (2022). Microbial contamination in waste collection: Unveiling this Portuguese occupational exposure scenario. Environ. Manag..

[40] Viegas C., Gomes B., Dias M., Carolino E., Caetano L.A. (2021). Occupational exposure to Aspergillus section Fumigati in Firefighter Headquarters. Microorganisms.

[41] Viegas C., Dias M., Almeida B., Aranha Caetano L., Carolino E., Quintal Gomes A., Twaruzek M., Kosicki R., Grajewski J., Marchand G. (2020). Are workers from waste sorting industry really protected by wearing Filtering Respiratory Protective Devices? The gap between the myth and reality. Waste Manag..

[42] Majchrzycka K., Okrasa M., Skóra J., Gutarowska B. (2016). Evaluation of the survivability of microorganisms deposited on filtering respiratory protective devices under varying conditions of humidity. Int. J. Environ. Res. Public Health.

[43] Moen B.E., Kayumba A., Sakwari G., Mamuya S.H.D., Bråtveit M. (2016). Endotoxin, dust and exhaled nitrogen oxide among hand pickers of coffee; a cross-sectional study. J. Occup. Med. Toxicol..

[44] Donlan R.M. (2002). Biofilms: Microbial life on surfaces. Emerg. Infect. Dis..

[45] Jankowska E., Reponen T., Willeke K., Grinshpun S.A., Choi K.J. (2000). Collection of fungal spores on air filters and spore reentrainment from filters into air. J. Aerosol. Sci..

[46] Maus R., Goppelsröder A., Umhauer H. (2001). Survival of bacterial and mold spores in air filter media. Atmos. Environ..

[47] Varga J., Baranyi N., Chandrasekaran M., Vágvölgyi C., Kocsubé S. (2015). Mycotoxin producers in the Aspergillus genus: An update. Acta Biol. Szeged..

[48] Sabino R., Veríssimo C., Viegas C., Viegas S., Brandão J., Alves-Correia M., Borrego L.M., Clemons K.V., Stevens D.A., Richardson M. (2019). The role of occupational Aspergillus exposure in the development of diseases. Med. Mycol..

[49] Segal B.H., Romani L.R. (2009). Invasive aspergillosis in chronic granulomatous disease. Med. Mycol..

[50] Patterson K., Strek M.E. (2010). Allergic bronchopulmonary aspergillosis. Proc. Am. Thorac. Soc..

[51] Shinohara N., Woo C., Yamamoto N., Hashimoto K., Yoshida-Ohuchi H., Kawakami Y. (2021). Comparison of DNA sequencing and morphological identification techniques to characterize environmental fungal communities. Sci. Rep..

[52] Kumsiri R., Kanchanaphum P. (2020). A Comparison of Four Molecular Methods for Detection of Aflatoxin-Producing Aspergillusin Peanut and Dried Shrimp Samples Collected from Local Markets around Pathum Thani Province. Thail. Sci..

[53] IARC (2012). International Agency for Research on Cancer. AFLATOXINS. https://monographs.iarc.who.int/wp-content/uploads/2018/06/mono100F-23.pdf.

[54] Viegas S., Viegas C., Oppliger A. (2018). Occupational Exposure to Mycotoxins: Current Knowledge and Prospects. Ann. Work Expo. Health.

[55] Viegas S., Viegas C., Martins C., Assunção R. (2020). Occupational Exposure to Mycotoxins—Different Sampling Strategies Telling a Common Story Regarding Occupational Studies Performed in Portugal (2012–2020). Toxins.

[56] Oldenburg M., Bittner C., Baur X. (2009). Health risks due to coffee dust. Chest.

[57] Brasel T.L., Martin J.M., Carriker C.G., Wilson S.C., Straus D.C. (2005). Detection of airborne Stachybotrys chartarum macrocyclic trichothecene mycotoxins in the indoor environment. Appl. Environ. Microbiol..

[58] Huttunen K., Korkalainen M., Viegas C., Viegas S., Quintal Gomes A., Taubel M., Sabino R. (2017). Microbial secondary metabolites and knowledge on inhalation effects. Exposure to Microbiological Agents in Indoor and Occupational Environments.

[59] Brera C., Caputi R., Miraglia M.C., Iavicoli I., Salerno A., Carelli G. (2002). Exposure assessmentto mycotoxins in workplaces: Aflatoxins and ochratoxin A occurrence in airborne dusts and human sera. Microchem. J..

[60] Lavicoli I., Brera C., Carelli G., Caputi R., Marinaccio A., Miraglia M. (2002). External and internal dose in subjects occupationally exposed to ochratoxin A. Int. Arch. Occup. Environ. Health..

[61] Mayer S., Curtui V., Usleber E., Gareis M. (2007). Airborne mycotoxins in dust from grain elevators. Mycotoxin Res..

[62] Mayer S., Viegas C., Pinheiro A.C., Sabino R., Viegas S., Brandão J., Verissimo C. (2015). Occupational exposure to mycotoxins and preventive measures. Environmental Mycology in Public Health: Fungi and Mycotoxins Risk Assessment and Management.

[63] Viegas S., Veiga L., Almeida A., Dos Santos M., Carolino E., Viegas C. (2016). Occupational Exposure to Aflatoxin B1 in a Portuguese Poultry Slaughterhouse. Ann. Occup. Hyg..

[64] Cherrie J.W., Semple S., Christopher Y., Saleem A., Hughson G.W., Philips A. (2006). How Important is Inadvertent Ingestion of Hazardous Substances at Work?. Ann. Occup. Hyg..

[65] Gorman Ng M., Davis A., van Tongeren M., Cowie H., Semple S. (2016). Inadvertent ingestion exposure: Hand- and object-to-mouth behavior among workers. J. Expo. Sci. Environ. Epidemiol..

[66] Verweij P.E., Snelders E., Kema G.H., Mellado E., Melchers W.J. (2009). Azole resistance in Aspergillus fumigatus: A side-effect of environmental fungicide use?. Lancet Infect. Dis..

[67] Snelders E., Camps S.M., Karawajczyk A., Schaftenaar G., Kema G.H., van der Lee H.A., Klaassen C.H., Melchers W.J.G., Verweij P.E. (2012). Triazole fungicides can induce cross-resistance to medical triazoles in Aspergillus fumigatus. PLoS ONE.

[68] Prigitano A., Venier V., Cogliati M., De Lorenzis G., Esposto M.C., Tortorano A.M. (2014). Azole-resistant Aspergillus fumigatus in the environment of northern Italy, May 2011 to June 2012. Euro Surveill..

[69] Verweij P.E., Chowdhary A., Melchers W.J., Meis J.F. (2016). Azole Resistance in Aspergillus fumigatus: Can We Retain the Clinical Use of Mold-Active Antifungal Azoles?. Clin. Infect. Dis..

[70] Berger S., El Chazli Y., Babu A.F., Coste A.T. (2017). Azole Resistance in Aspergillus fumigatus: A Consequence of Antifungal Use in Agriculture?. Front. Microbiol..

[71] Morton V., Staub T. (2008). A Short History of Fungicides. The American Phytopathological Society. https://www.apsnet.org/edcenter/apsnetfeatures/Pages/Fungicides.aspx.

[72] Azevedo M.M., Faria-Ramos I., Cruz L.C., Pina-Vaz C., Rodrigues A.G. (2015). Genesis of azole antifungal resistance from agriculture to clinical settings. J. Agric. Food Chem..

[73] Price C.L., Parker J.E., Warrilow A.G., Kelly D.E., Kelly S.L. (2015). Azole fungicides—Understanding resistance mechanisms in agricultural fungal pathogens. Pest. Manag. Sci..

