# Supplementary material for: Microbial Contamination in the Coffee Industry: An Occupational Menace besides a Food Safety Concern?
Source: Int J Environ Res Public Health. 2022 Oct 18;19(20):13488. doi: 10.3390/ijerph192013488 (PMC9602572; doi:10.3390/ijerph192013488)
Supplement: Supplementary file 1 [file ijerph-19-13488-s001.zip › ijerph-1957588-supplementary.pdf]

Supplementary material

Table S1 - Shannon and Simpson diversity indices in the EDC matrix

Industry	Species	Media	Diversity indices	
		DG18 ($CFUm^{-2}day^{-1}$)	Shannon Index (H)	Simpson Index (D)
A	*Penicillium* sp.	1981.599	1.34	2.35
	Cladosporium sp.	346.780		
	A. section *Nigri*	304.317		
	A. section *Circumdati*	208.776		
	Mucor sp.	113.234		
	A. section *Fumigati*	49.540		
	Lichtheimia sp.	46.001		
	Chrysosporium sp.	28.309		
	Paecilomyes sp.	21.231		
	A. section *Flavi*	17.693		
	A. section *Nidulantes*	17.693		
	A. section *Aspergilli*	10.616		
B	*Penicillium* sp.	5046.001	1.23	2.83
	A. section *Circumdati*	3336.872		
	Cladosporium sp.	1641.897		
	A. section *Nigri*	318.471		
	A. section *Nidulantes*	77.849		
	A. section *Terrei*	35.386		
	A. section *Aspergilli*	31.847		
	F. verticilloides	28.309		
	A. section *Flavi*	24.770		
	Chrysosporium sp.	14.154		
	F.graminearum	10.616		

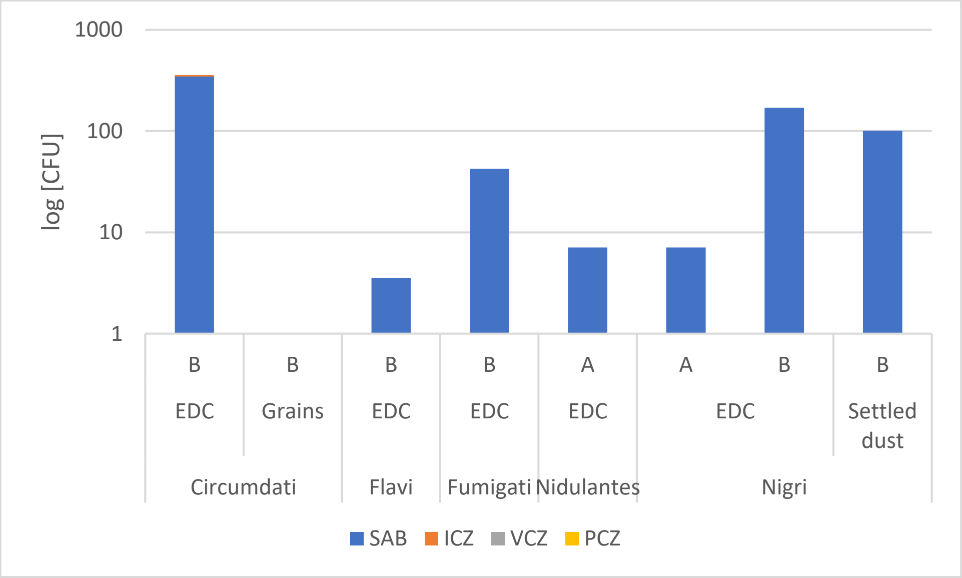

Figure S1 – *Aspergillus* sections' frequencies, per industry (A, B) and sampling matrix (EDC, grains, settled dust), by screening in azole-supplemented Sabouraud dextrose agar (SDA) media. ICZ, 4mg/ml itraconazole; VCZ, 2 mg/ml voriconazole; PCZ, 0.5 mg/ml posaconazole.

Table S2 – *Aspergillus* sections detection in the different matrices analysed

Aspergillus sections	Matrix	Industry	CFU.m^{-2} (MEA/DG18)	C_q
Nidulantes	EDC	B	0/0	33.19
			0/0	29.53
		A	0/0	31.39
			0/0	34.65
			0/0	33.98
			0/0	36.22
	Coffee beans	B	0/0	39.42
		A	0/0	37.06
			0/0	38.35
	FRPD	B	0/0	38.19
	Settled dust	B	0/0	37.66
			0/0	38.96
		A	0/0	39.22
			0/0	36.34
			0/0	33.49
			0/0	36.75
			0/0	35.88
			0/0	34.94
			0/0	38.19
			0/0	39.99
Circumdati	EDC	B	0/0	34.22
			0/14.153	33.82
			0/14.15	32.4
			0/24.77	36.47
			0/74.30	36.29
			424.63/2689.310	30.59
			84.93/0	31.16
			7.07/0	32.49
			0/0	33.3
			0/0	33.91
			17.69/0	34.18
			0/24.77	33.83
			0/14.15	32.06
			0/46.00	34.68
			0/141.54	33.42
			0/13.27	27.71
		A	0/0	35.47
			0/0	37.31
			0/0	35.54
			0/0	36.08
	Coffee beans	A	0/0	35.19
	Settled dust	B	0/0	34.67
		A	0/0	33.99
			0/0	34.14

	0/0	34.52
	0/0	34.5

S2).

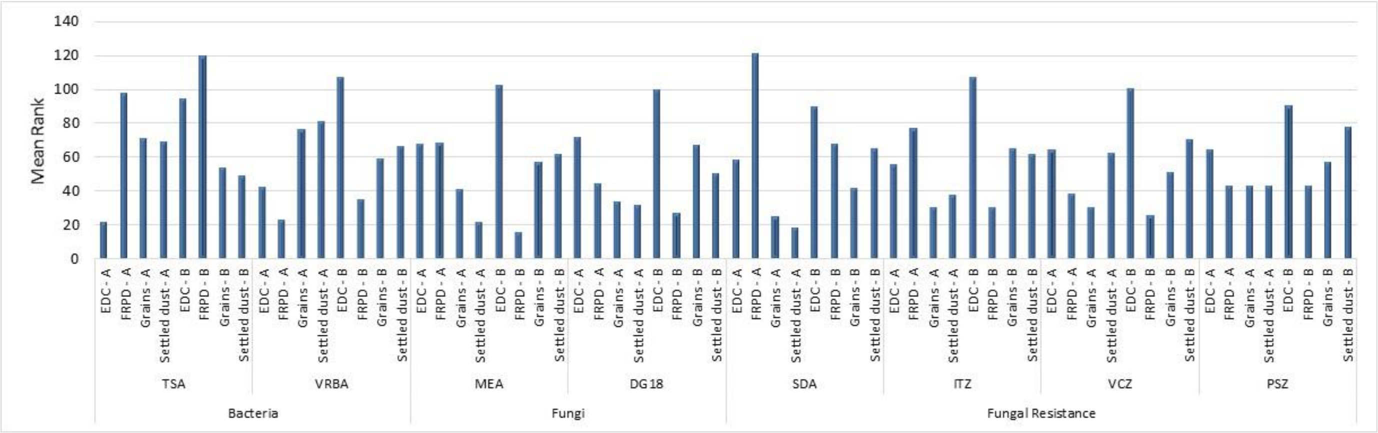

Figure S2 - Comparison of bacterial, fungal contamination and fungal resistance between the sampling methods of the two industries (A and B). Results of the Kruskal Wallis test
